# Mixing Controlled Adsorption at the Liquid-Solid Interfaces in Unsaturated Porous Media

**DOI:** 10.1007/s11242-022-01747-x

**Published:** 2022-02-05

**Authors:** Ishaan Markale, Andrés Velásquez-Parra, Andrés Alcolea, Joaquín Jiménez-Martínez

**Affiliations:** 1 Department of Water Resources and Drinking Water, Eawag, 8600 Dübendorf, Switzerland; 2 Department of Civil, Environmental and Geomatic Engineering, ETH Zurich, 8093 Zürich, Switzerland; 3 HydroGeoModels AG, Tösstalstrasse 23, 8400 Winterthur, Switzerland

**Keywords:** Pore-scale adsorption, Solute mixing, Dilution, Phase saturation, Isotherm functional shape, Surfactants

## Abstract

The unsaturated zone, located between the soil surface and the phreatic level, plays an important role in defining the fate of any substance entering the subsoil. In addition to the processes of flow and transport taking place in the liquid phase, surface reactions such as adsorption to the solid phase may occur and increase the residence time of the substance entering the system. In this study, we aim to understand the pore-scale mechanisms that control adsorption in unsaturated systems. We combine 2D pore-scale experimental images with numerical simulations to analyze flow, transport, and adsorption under different liquid saturation degrees. We demonstrate the role of mixing on adsorption at the liquid-solid interfaces by analyzing the deformation in time of a pulse-injected surfactant. We also analyze the impact of the isotherm functional shape and the inclusion of the liquid-gas interfaces as adsorption sites on this surface reaction. The enhancement of mixing as saturation decreases is accompanied by a reduction in the amount of adsorbed mass, located mainly along preferential flow paths, where the solute is primarily transported. For the same isotherm, a nonlinear behavior of adsorption as a function of liquid saturation has been observed. This is explained by the nonlinear variation of the volume fraction of liquid behaving as preferential path or stagnation zone as liquid saturation decreases, despite the linear decrease in the surface area of solids accessible for adsorption.

**Supplementary Information:**

The online version contains supplementary material available at 10.1007/s11242-022-01747-x.

## Article Highlights

- Pore-scale adsorption is reduced as water saturation decreases because of faster solute dilution.
- Isotherm coefficient controls the magnitude of adsorbed mass and isotherm exponent controls the temporal evolution of adsorption at pore-scale.
- Flow channeling and incomplete mixing at pore-scale result in nonlinear behavior of adsorbed mass with varying water saturation.

**Supplementary Information:**

The online version contains supplementary material available at 10.1007/s11242-022-01747-x.

## Introduction

Chemical reactions in natural porous media are important in several subsurface applications, such as groundwater management (Sebilo et al. 2013), contaminant (bio-)remediation (Williams et al. 2009), and risk assessment in underground waste storage (Winogard 1981), among others. One of the most widespread reactions in porous media is adsorption. It is defined as the accumulation of molecules of an adsorbate at the interfaces with other material phases in the system, namely at the liquid-solid and also at the liquid-gas interfaces in unsaturated porous media, i.e., with the presence of an immiscible phase within the pore space. The inherent heterogeneity of porous media impacts adsorption dynamics, as it controls the accessibility of the bulk solution to different regions of the system. This can be observed, e.g., in the uneven distribution of adsorption between a pore body and a pore throat (i.e., at the point of shortest distance between neighboring grains) (Skopp 2009). Such spatial variability in the adsorption to solid surfaces is magnified by the additional presence of an immiscible phase in the pore space, which enhances the heterogeneity of the system, reflected in the more heterogeneous fluid flow structure (Velásquez-Parra et al. 2021), and increases the dynamics of solute transport (Jiménez-Martínez et al. 2017).

Surfactants (synthetic chemicals) belong to some of the interfacial partitioning contaminants more often found in the subsoil. They are usually present at low concentrations, which renders their detection difficult. They are mostly used in the formulation of pesticides and personal care products, among many others, being their main use the formulation of detergents. Surfactants enter the subsoil through diffuse and/or punctual sources of agricultural, urban, and industrial origin. Their presence in the subsoil has been extensively studied (Eichhorn et al. 2002; Jonkers et al. 2003). It has been observed that they tend to persist at very low concentrations even after the implementation of treatment techniques (Mcavoy et al. 1998). Some studies have also used synthetic chemicals (in liquid and gas phase), including dyes, as tracers to characterize the adsorption process in both fully and partially saturated systems, at both continuum (i.e., Darcy-scale) (Kim et al. 1997; Costanza and Brusseau 2000; Vanderborght et al. 2002; Hillebrand et al. 2015) and pore-scale (Zhang et al. 2008; Ryan et al. 2011; McDonald et al. 2016; Vanson et al. 2017).

Surfactants can be further affected by other processes such as degradation caused by light or microorganisms, which deplete their environmental concentration (Bork et al. 2020). A general overestimation of adsorption from laboratory batch experiments when compared with field scale experiments, i.e., infiltration experiments, has also been observed (Botella-Espeso et al. 2021). However, the role that solute transport processes, and in particular mixing, which increases the volume occupied by the solute by smoothing out concentration gradients (Ottino 1989; Kitanidis 1994; Dentz et al. 2011), plays on adsorption has received far less attention, especially at the pore-scale, where the interaction between distinct phases can be directly assessed.

It has been previously shown that unsaturated conditions allow the development of channelized flow, in which preferential paths and stagnation zones of high and low liquid flow velocity, respectively, develop in the system (De Gennes 1983; Holzner et al. 2015). Preferential paths reduce the residence time of a solute in the porous medium, shortening the time available for it to mix with the resident solution (Gouet-Kaplan and Berkowitz 2011). However, they can also sustain large concentration gradients transverse to the mean flow direction, increasing the efficiency of the mixing process along them (Jiménez-Martínez et al. 2015). At the same time, this nonuniform flow field of regions of contrasting high and low velocities causes the solute to spread and mix in a radically different manner than in an uniform flow field (Dentz et al. 2011; Jiménez-Martínez et al. 2017). This leads to the so-called incomplete mixing behind the solute front, even for times longer than the diffusive time (with either the pore length or the pore throat width as characteristic length) (Paster et al. 2015; Wright et al. 2017). Both flow channeling and incomplete mixing can thus impact fluid-solid reactions, namely adsorption. In a recent work of de Vries et al. (2021) adsorption of colloids at the liquid-solid interfaces in a fully saturated porous medium has been described at pore scale. However, to our knowledge, a systematic study of the impact of the saturation degree (i.e., volume fraction of pore space occupied by water) on solute mixing and of its consequences for adsorption at the solid surfaces has not been carried out yet.

The impact of the saturation degree and the control of solute mixing on adsorption is difficult to assess experimentally at the pore-scale. To fill this gap in the literature, we address numerically the study of pore-scale adsorption of a surfactant using *quasi* 2D solute transport simulations of a porous medium at four different saturation degrees. These are created from experimental images of a milli-fluidics setup (Jiménez-Martínez et al. 2017). We study the control of surfactant mixing on adsorption using a pulse injection with no impact on the velocity field. This type of injection is ideally suited for understanding mixing and adsorption dynamics since it is commonly found in nature and it allows for a better estimation of analytical scaling laws of both dispersion and mixing. We first simulate steady-state flow in the liquid phase (no multiphase flow) for all four saturation degrees, adding in a second step both surfactant transport processes and adsorption at the liquid-solid interfaces. The flow rate was adapted to have the same macroscopic Péclet number for each individual case. For adsorption, we adopt a Freundlich isotherm and employ physical parameters reported for surfactants in soils with low contents of clay and organic matter (Chien-Hsiang and Elias 1995; Botella-Espeso et al. 2021). Finally, we analyze the impact of different isotherm parameterizations and of adsorption at the liquid-gas interfaces on the response of the system.

This paper is organized as follows. Section 2 summarizes the experimental data set (i.e., the four saturation degrees), and the numerical model adopted for solving flow, solute transport, and adsorption. In Sect. 3, we present the results of the transport simulations, and we describe the control of both mixing and concentration dilution on adsorption at the pore-scale, as well as the impact of the isotherm parameterization and of adsorption at the liquid-gas interfaces on the total adsorbed mass. In Sect. 4, we discuss the results, explaining the observed nonlinear behavior of adsorption as a function of the saturation degree.

## Methodology

### Experimental Images of Saturation Degrees

**Fig. 1:**
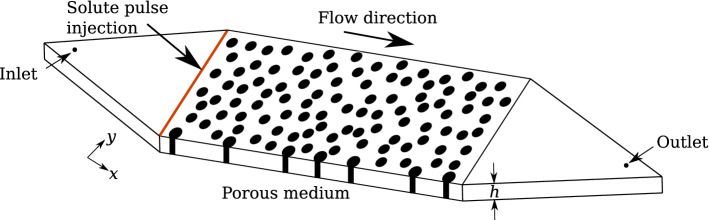
Scheme of the experimental setup from Jiménez-Martínez et al. (2017) used to build up the fluid flow and reactive transport numerical model. It displays the location of the inlet, where a constant flow rate of water only is imposed; of the outlet, where atmospheric pressure is set; and of the solute pulse (red line) to be injected. *h* corresponds to the thickness (i.e., vertical gap between the upper and lower plates) of the *quasi* 2D experimental porous medium

Our numerical simulation analyses are based on two-dimensional images of a water-wet porous medium at four different saturation degrees ($S_{\mathrm {w}}= 1.00$, 0.83, 0.77, and 0.71). Such images were obtained after the milli-fluidic experiments of Jiménez-Martínez et al. (2017). The porous medium, with dimensions $132\,\mathrm {mm}\times 128\,\mathrm {mm}$ ($3982 \times 2182$ pixels; pixel size of $0.04\,\mathrm {mm}$) and a vertical gap $h = 0.5\,\mathrm {mm}$, consists of solid circular obstacles (which mimic solid grains) with a mean diameter $d=0.83\pm 0.22\,\mathrm {mm}$ irregularly distributed across the geometry, resulting in a final porosity $\phi =0.71$ and permeability $\kappa =7.8 \times 10^{-9}$ m$^{2}$ (see Fig. 1). The bulk density of the porous medium is $\rho _\mathrm {b}=783\,\mathrm {kg\,m^{-3}}$. The average pore body size is $\lambda = 1.85\,\mathrm {mm}$ and the average pore throat is $a = 1.17\,\mathrm {mm}$. The unsaturated cases were obtained by injecting air and water simultaneously until a steady-state and equal bulk saturation was achieved in both longitudinal and transverse directions. The simultaneous injection of water and air was then stopped resulting in a static distribution of phases (recall Figure S1 in the Supporting Information, which includes the spatial distribution of solid, liquid, and gas phases for all four saturation degrees).

### Fluid Flow Modeling

We simulated flow, solute transport, and adsorption using the finite element software COMSOL Multiphysics®. Models were built upon the experimental images obtained for each $S_{\mathrm {w}}$. Mesh elements were all smaller than the pixel size (approximately $900'000$ elements). Steady-state velocity fields were solved in the liquid phase. i.e., the gas phase remains immobile, by applying the incompressible Navier-Stokes formulation for the simplified case of Stokes flow (Eqs. 1 and 2 ), in which the flow around grains and air clusters is only controlled by viscous forces. This is given by

1 $$\begin{aligned} \nabla \cdot u = 0, \end{aligned}$$

2 $$\begin{aligned} \mu \cdot \nabla ^2u -\nabla p + F =0, \end{aligned}$$

where *u* $\mathrm {[m\,s^{-1}]}$ is the liquid flow velocity, *p* $\mathrm {[kg\,m^{-1}s^{-2}]}$ is the liquid pressure, $\mu = 1\times 10^{-3}\,\mathrm {kg\,m^{-1}s^{-1}}$ is the dynamic viscosity of water, and where $F = u\cdot \mu /\kappa _{\mathrm {h}}$ $\mathrm {[kg\,m^{2}s^{-2}]}$ is an additional term to account for the effect of the third dimension on the depth-averaged velocity. This additional Darcy-like term accounts for the drag force exerted on the liquid by the upper and lower plates of the reference experiment (Jiménez-Martínez et al. 2017). $\kappa _{\mathrm {h}}$ is the permeability and can be approximated as $\kappa _{\mathrm {h}} \sim h^2/12$ (Ferrari et al. 2015; Soulaine et al. 2021).

Equations 1 and 2 were solved for each saturation degree (i.e., for each distribution of phases, Figure S1) subject to the following boundary conditions. A constant flow rate of water only was specified at the inlet (Fig. 1), while atmospheric pressure was set at the outlet. Liquid-solid interfaces were treated as no-slip boundaries, while a slip boundary condition was set at the liquid-gas interfaces. The injection flow rate *Q* (see Table 1) was chosen so that all interfaces remained immobile and non-deformed, i.e., the flow of the water does not disturb the established geometry of the air clusters. This is confirmed by a maximum capillary number $C_{\mathrm {a}} = F_{\mathrm {v}}/F_{\mathrm {c}}=0.013$ (for $S_{\mathrm {w}}=0.71$ at the flow rate reported in 1), where $F_{\mathrm {v}}$ are the viscous forces and $F_{\mathrm {c}}$ is the capillary resistance, following Tang et al. (2019).

### Transport and Adsorption Modeling

**Fig. 2:**
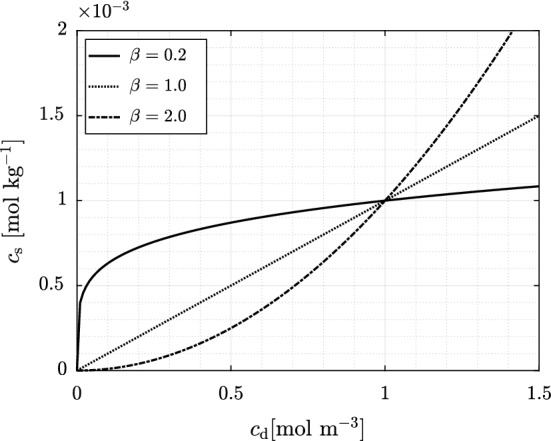
Freundlich adsorption isotherm showing the relationship between $c_\mathrm {s}$ and $c_\mathrm {d}$ for three different exponents $\beta =0.2,\,1.0$ and 2.0, all of them computed with a constant coefficient $K_\mathrm {d}$ $= 1\,\mathrm {m^{3}\,kg^{-1}}$

The steady-state velocity field of the liquid computed for each saturation degree was used to set up a transient transport model, solving for advection, diffusion, and adsorption. The following transport equation was employed:

3 $$\begin{aligned} \frac{\partial \phi c_\mathrm {d}}{\partial t}+\frac{\partial \rho _\mathrm {b} c_\mathrm {s}}{\partial t}=\nabla (D\nabla c_\mathrm {d}) - u\cdot \nabla c_\mathrm {d} + r \end{aligned}$$

where $c_\mathrm {d}$ $\mathrm {[mol\,m^{-3}]}$ corresponds to the solute concentration in the dissolved (liquid) phase, $c_\mathrm {s}$ $\mathrm {[mol\,kg^{-1}]}$ is the adsorbed concentration in the solid phase, $D\,[\mathrm {m^{2}\,s^{-1}}]$ is the molecular diffusion coefficient, and $r\,[\mathrm {mol\,m^{-3}\,s^{-1}}]$ is the reaction rate of the solute species (i.e., sink term). A temporal discretization based on a geometric progression with common ratio 1.02, a starting time increment of $0.0023\,\mathrm {s}$ and a total of $2'500$ time steps was defined to solve the transient analysis, which spans over $10'000\,\mathrm {s}$. The top and bottom boundaries along with the liquid-gas interface are set as no flux boundaries, i.e., $\partial c_\mathrm {d}/\partial n = 0$, where *n* is the normal direction to the boundary. The temporal evolution of adsorption-desorption, i.e., the solute mass exchange to or from the solid phase, respectively, is defined by expressing the solute concentration in the solid phase $c_\mathrm {s}$ as a function of the concentration in the liquid phase $c_\mathrm {d}$, i.e., isotherm. As transport takes place within the porous medium, species might attach to (adsorption) and detach from (desorption) the solid phase, increasing or reducing $c_\mathrm {s}$, respectively, thus affecting the concentration of the solute transported through the system. Depending on the isotherms describing the adsorption and the desorption processes, the transport of the solute in the porous medium can thus be slowed down or enhanced. We can relate $c_\mathrm {d}$ to $c_\mathrm {s}$ by the so-called Freundlich isotherm, expressed as

4 $$\begin{aligned} c_\mathrm {s} = \frac{1}{\rho _\mathrm {b}\,K_\mathrm {d}\,M_\mathrm {w}} \left( \frac{c_\mathrm {d}}{c_\mathrm {ref}} \right) ^{\beta }, \end{aligned}$$

where $K_\mathrm {d}$ $\mathrm {[m^{3}\,kg^{-1}]}$ is the isotherm coefficient, $M_\mathrm {w}\,\mathrm {[kg\,mol^{-1}]}$ is the molecular weight of the adsorbate, $c_\mathrm {ref}\,\mathrm {[mol\,m^{-3}]}$ is a reference concentration used for units correction, and $\beta$ (dimensionless) is the isotherm exponent. $K_\mathrm {d}$ and $\beta$ depend on factors such as the type of adsorbent and adsorbate, temperature, and pH, among others (Skopp 2009; Bork et al. 2020). Previous studies (Brownawell et al. 1997; John et al. 2000; Botella-Espeso et al. 2021) proved that the Freundlich isotherm (with different $K_\mathrm {d}$ and $\beta$ values) is the most suitable isotherm for reproducing adsorption of surfactants in soils, specially for the case of non-ionic surfactants. We simulate three different isotherm exponents $\beta =0.2,\, 1.0$ and 2.0, and three different isotherm coefficients $K_\mathrm {d}$ (spanning over a range of three orders of magnitude, from $10^{-1}$ to $10^{1}\,\mathrm {m^{3}\,kg^{-1}}$), all of which are representative for soils with low contents of clay and organic matter (Botella-Espeso et al. 2021). Figure 2 displays the adsorption isotherm for different exponents $\beta$ and an equal and constant coefficient $K_\mathrm {d}$ $\,= 1\,\mathrm {m^{3}\,kg^{-1}}$. A molecular weight $M_\mathrm {w}=0.7\,\mathrm {kg\,mol^{-1}}$ is adopted, which lies within the range of values for most commercial surfactants [$0.3-0.7\,\mathrm {kg\,mol^{-1}}$] (Rodriguez et al. 2005). Further details on these parameters are provided in Sect. 3.3. Hysteresis in the adsorption and desorption processes has been observed in natural soils (Skopp 2009). However, for the purpose of this study, we only account for adsorption at the liquid-solid interfaces (and the impact of adsorption at the liquid-gas interface) and assume that no desorption takes place.

A pulse (Dirac) injection of $c_{\mathrm {d,0}} = 1.0 \, \mathrm {mol\,m^{-3}}$ is applied at $t=0\, \mathrm {s}$ uniformly along the left boundary (Fig. 1). The same injection condition was used for all four saturation degrees and adsorption isotherms considered. A constant flow rate $Q=2.77 \times 10^{-8}\, \mathrm {m^3s^{-1}}$ for the fully saturated case ($S_{\mathrm {w}}= 1.00$) results in a Péclet number (i.e., ratio of advective to diffusive transport) $\mathrm {Pe} = {\bar{u}}a^2/2D\lambda =4.42$, where ${\bar{u}}$ is the average flow velocity. A molecular diffusion coefficient $D\,=\,8 \times 10^{-10}$ $\mathrm {m^2s^{-1}}$ is adopted, which is well within the range of values measured experimentally [$2\times 10^{-10}-10 \times 10^{-10}$ $\mathrm {m^2s^{-1}}$] (Lucassen and Giles 1975; Weinheimer et al. 1981; Song et al. 2006). The advective time scale over a pore size is estimated as $t_a=\lambda /{\bar{u}}$ [s] and the diffusive time scale over a pore throat as $t_d=a^2/2D$ [s]. In order to study the dependency of the numerical results on the saturation degree only, we keep the same $\mathrm {Pe}$ for all analyzed $S_{\mathrm {w}}$ by adjusting the flow rate accordingly (Table 1). The relative importance of advection to adsorption is measured by the Damköhler number (Da), representing the ratio of advection ($t_a$) to reaction ($t_r$) timescales, ${\mathrm {Da}}=t_a/t_r$. The reaction timescale is defined by the kinetics of adsorption $\varepsilon \,\mathrm {[s^{-1}]}$ for a first-order reaction under well-mixed conditions $t_r=1/\varepsilon \,[\mathrm {s}]$ (Botella-Espeso et al. 2021), where an instant equilibrium between $c_{\mathrm {d}}$ and $c_{\mathrm {s}}$ is assumed. The adsorption is fast compared to advection, ${\mathrm {Da}}=t_a/t_r\gg 1$. We verify mass conservation in time by analyzing the sum of concentrations found in the liquid and solid phases, which has to remain constant before breakthrough. The total mass adsorbed in the solid phase over the entire domain $M_\mathrm {s}$ $\mathrm {[mol\,kg^{-1}]}$ is calculated at each time step and compared for the different saturation degrees $S_{\mathrm {w}}$ and adsorption parameters $\beta$ and $K_\mathrm {d}$. We assume that the geometry of the porous medium remains unchanged after the process of adsorption i.e., the pore size distribution, permeability and porosity are constant.

**Table 1:** Summary of flow conditions imposed in the 2D numerical simulation for each saturation degree $S_\mathrm {w}$. *Q* is the prescribed flow rate for the same Péclet number $\mathrm {Pe}=4.42$, ${\bar{u}}$ is the average pore velocity, *Re* is the Reynolds’ number and $t_a$ is the advective time. $\Gamma _{\mathrm {l-s}}$ is the length of the liquid-solid interface and $\Gamma _{\mathrm {l-g}}$ is the length of liquid-gas interface

$S_\mathrm {w}$	*Q* $\mathrm {[m^3s^{-1}]}$	${\bar{u}}$ $\mathrm {[m\,s^{-1}]}$	*Re*	$t_a$ [s]	$\Gamma _{\mathrm {l-s}}$ $\mathrm {[mm]}$	$\Gamma _{\mathrm {l-g}}$ $\mathrm {[mm]}$	$\Gamma _{\mathrm {l-g}}/\Gamma _{\mathrm {l-s}}$
1.00	2.770$\times 10^{-8}$	9.56$\times 10^{-6}$	0.0112	192.9	13492.6	–	–
0.83	2.088$\times 10^{-8}$	9.54$\times 10^{-6}$	0.0112	193.9	11426.9	3449.7	0.30
0.77	1.792$\times 10^{-8}$	9.52$\times 10^{-6}$	0.0111	194.3	10720.6	3812.7	0.36
0.71	1.501$\times 10^{-8}$	9.51$\times 10^{-6}$	0.0111	194.5	10195.8	4540.2	0.45

### Quantification of Mixing and Dilution

Solute transport in porous media is mainly ruled by the nature and heterogeneity of the flow field and by local mass transfer, both of which control the shape and evolution of the injected pulse. A number of different metrics have been proposed to quantify mixing and dilution (Dentz et al. 2011). We recall the definition of mixing zone ${\Omega }_\mathrm {m}$, i.e., the area in the pore space at a particular instant in time where concentration gradients between the injected and resident solutions exist and are smoothed out in time. For the considered pulse injection, this zone also corresponds to the portion of the pore space where solute adsorption to the solid phase can occur. The mixing zone ${\Omega }_\mathrm {m}$ can be characterized by its area $A_\mathrm {m}\mathrm {[m^{2}]}$, in which $0< c_{\mathrm {d}}(x,y) < 1$. In our study, this is implemented numerically as the area $A_\mathrm {m}=\{(x,y)|0.02<c_\mathrm {d}(x,y)<0.98)\}$ to remove spurious numerical effects. In addition, we also evaluate the temporal evolution of the mean concentration within the mixing zone, i.e., the dilution, ${\overline{c}}_{{\Omega }_\mathrm {m}}(t)=\sum _{\varOmega _\mathrm {m}}^{}c_{\mathrm {d}}(x,y;t)/A_\mathrm {m}(t)$.

## Results and Discussion

### Impact of Saturation on Transport and Mixing

**Fig. 3:**
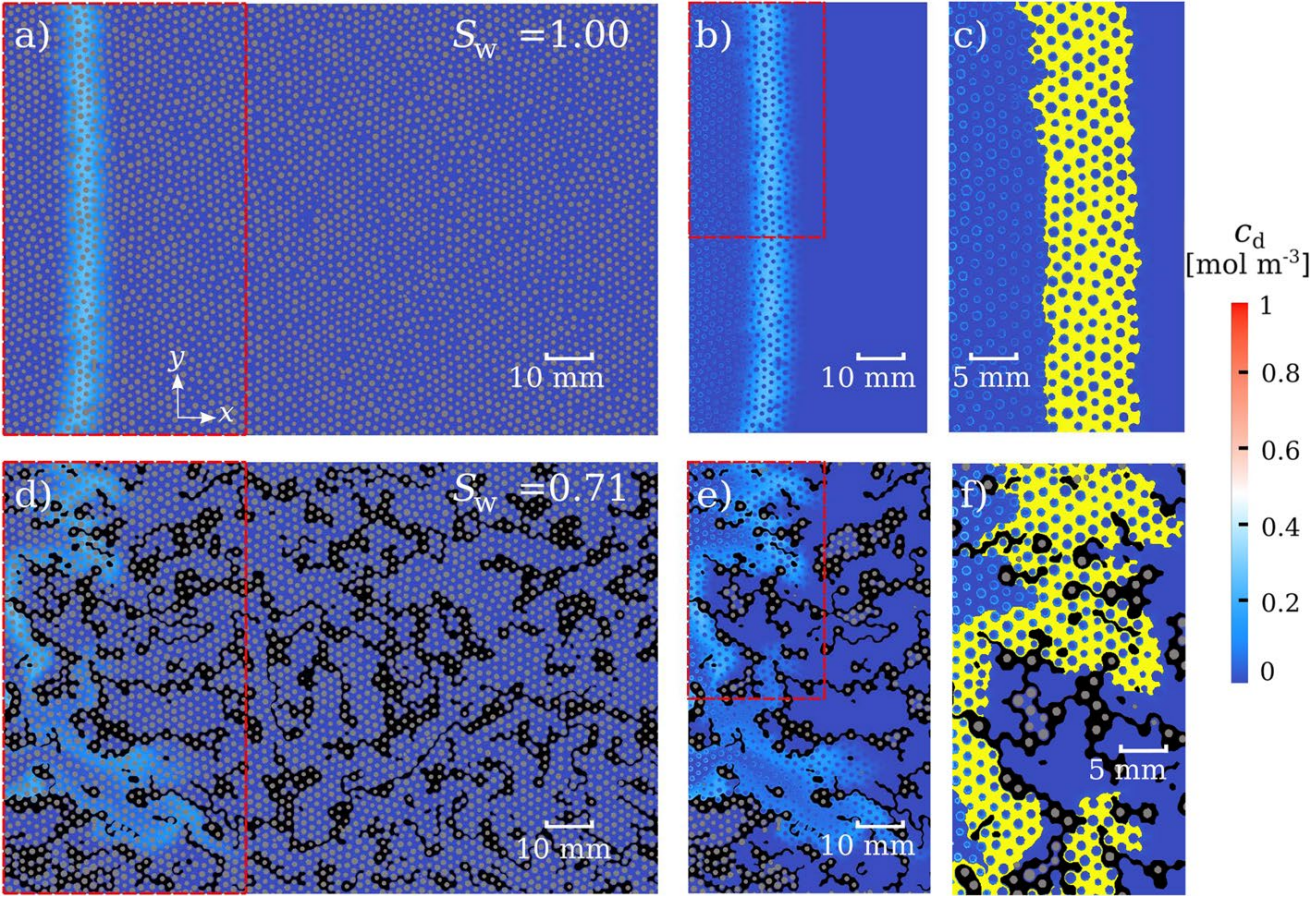
Concentration field for saturation degrees $S_{\mathrm {w}}=1.00$ (panels a-c) and $S_{\mathrm {w}}=0.71$ (panels d-f) at a common dimensionless time $t/t_a=10$ and under the same Péclet number. The air is depicted in black, whereas the solid grains are depicted in gray. Panel d shows the preferential flow paths generated by the presence of air, i.e., the fingering effect (see Supporting Information, Figure S1 for the preferential paths and stagnation zones of the velocity field for all $S_{\mathrm {w}}$). Panels b and e display a close up to the red inset in panels a and d. Part of the gray mask (within the connected liquid phase) has been removed in panels b-c and e-f, which renders a better image of the solute adsorbed to the solid grains. Finally, panels c and f (a close up to the red inset in panels b and e) display the mixing volume ${\Omega }_{\mathrm {m}}$ in yellow, which clearly showcases the effect of the system heterogeneity on the stretching of the injected solute pulse. Spatio-temporal evolution of transported solute (Movies M1-M4) and adsorption and of the mixing volume (Movies M5-M8) for all $S_{\mathrm {w}}$ can be found in the Supporting Information

**Fig. 4:**
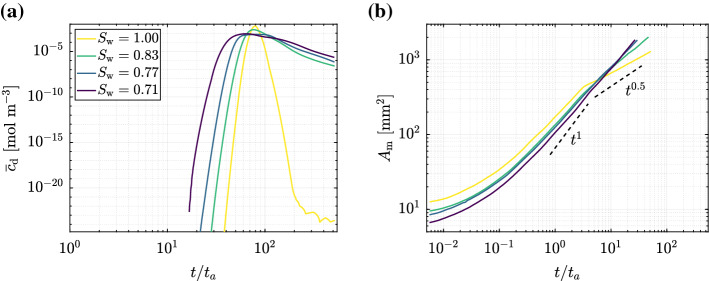
**a** Breakthrough curves (BTCs) computed at the outlet of the system for a conservative solute traveling through the pore space after a pulse injection, and **b** temporal evolution of mixing, as quantified by the mixing area $A_\mathrm {m}$, for the four different saturation degrees analyzed $S_{\mathrm {w}}=1.00,\,0.83,\,0.77$, and 0.71. The Péclet number $\mathrm {Pe}=4.42$ has been kept constant for all cases. The scalings $t^1$ and $t^{0.5}$ represent a ballistic and a Fickian regime, respectively, and are shown for visual reference. Time has been non-dimensionalized by the advective time $t_a$

Figure 3 displays the spatial distribution of solute concentration and the mixing zone for $S_\mathrm {w}$ $=1.00$ (Fig. 3a–c) and $S_\mathrm {w}$ $=0.71$ (Fig. 3d–f) for a common dimensionless time $t/t_a=10$ and same $\mathrm {Pe}$. Concentration fields of the solute reveal the impact of saturation degree on solute transport, i.e., different spreading and development of distinct fingering patterns (Fig. 3a and d). This impact is best evaluated quantitatively by computing the breakthrough curves (BTCs) and the temporal evolution of the mixing area. Figure 4a depicts the BTCs for all $S_{\mathrm {w}}$, showing the average solute concentration in the liquid phase ${\overline{c}}_\mathrm {d}$ at the right edge of the porous domain. BTCs clearly depict the impact of the immiscible phase (air) and of the heterogeneity it adds in the system on solute transport across saturations. The solute shows larger spreading, with earlier arrival times and a more pronounced tailing (i.e., longer residence times) as $S_{\mathrm {w}}$ decreases. These two effects are caused by preferential paths of high velocity and stagnation zones of contrasting low velocities, respectively, both of which become more pronounced at lower $S_{\mathrm {w}}$ (Wildenschild et al. 2002; Bromly and Hinz 2004; Karadimitriou et al. 2016; Triadis et al. 2019; Velásquez-Parra et al. 2021). Refer to Figure S1 in the Supporting Information for a visualization of the velocity fields in the liquid phase. The temporal evolution of the area of the mixing zone $A_\mathrm {m}$, as defined in Sect. 2.4, is plotted in Fig. 4b for all $S_{\mathrm {w}}$. As previously observed, $A_\mathrm {m}$ rises sharply at early times as the solute starts to invade the pore space. This is depicted by a non-Fickian, almost ballistic behavior, with $A_{\mathrm {m}}\sim t^1$ regardless of saturation degree. The sharp increase of $A_\mathrm {m}$ reflects the dominant role of dispersion on solute transport at early times, while the line pulse gets stretched on its travel through the pore space. Smaller magnitudes of $A_\mathrm {m}$ for the unsaturated cases at these early times respond to the reduced pore space available for flow caused by the size and spatial distribution of air clusters near the inlet (see Figure S1 in the Supporting Information). At intermediate times $(t/t_a=1)$, the mixing area growth still scales faster than Fickian $(t^1)$. For the duration of the simulations, a transition toward a Fickian condition $(t^{0.5})$ is only observed for the fully saturated case at around $t/t_a=3.0$. This is caused by the diffusion-controlled coalescence of fingers formed at early times, as developed theoretically (Le Borgne et al. 2013) and observed experimentally (de Anna et al. 2014; Jiménez-Martínez et al. 2015; Markale et al. 2021). This transition is usually referred to as mixing time, after which the porous medium behaves as a diffusive system with regards to liquid mixing. No transition to a Fickian behavior was observed for $S_\mathrm {w}$ $<1.00$, although a slight reduction in the scaling for $S_{\mathrm {w}}=0.83$ and $S_{\mathrm {w}}=0.77$ at late times ($t/t_a >10$) was obtained. The conservation of non-Fickian temporal scalings for the unsaturated cases indicates further solute spreading and incomplete mixing as a result of the increased system heterogeneity, i.e., as saturation degree decreases.

### Mixing and Dilution Driven Pore-Scale Adsorption

We now demonstrate how mixing, and dilution within the mixing zone, actually controls adsorption in porous media as function of the saturation degree. Figure 3 shows the travel of the solute pulse through the pore space, where adsorption takes place at the liquid-solid interfaces. Besides the solute spreading (which depends on saturation) described in Sect. 3.1, dilution within the mixing zone also starts happening right after injection, reducing the concentration of the injected pulse ($c_{\mathrm {d}}=1.0\,\mathrm {mol\,m^{-3}}$) as it moves through the system. In general, the amount of adsorbed solute right behind the front reduces in the flow direction as lesser mass and diluted concentration in the liquid phase remains available for adsorption to occur (see Movie M1 in the Supporting Information for the spatio-temporal evolution of adsorption). While at $S_{\mathrm {w}}=1.00$ the solute pulse travels without major deformation, allowing adsorption to occur at all available solid grain surfaces (Fig. 3a and b), for the unsaturated case $S_{\mathrm {w}}=0.71$ most of the solute is transported along preferential paths (see Figure S1 in the Supporting Information for a visualization of the velocity field), where most of the adsorption takes place (Fig. 3d and e). The strong flow channeling can even cause that some pore space areas enclosed by neighboring preferential paths show only little adsorption in spite of not belonging to stagnation zones *per se*. Adsorption can also take place in the stagnation zones where the solute is mainly subjected to diffusion, although it occurs at a much slower rate compared to regions whose flow is described by preferential paths.

**Fig. 5:**
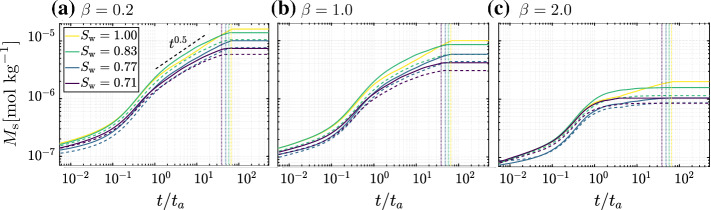
Temporal evolution of the absorbed mass in the solid phase $M_\mathrm {s}$  for three different adsorption regimes characterized by isotherm exponents a) $\beta =0.2$ b) $\beta =1.0$ and c) $\beta =2.0$. The same isotherm coefficient $K_\mathrm {d}$= $1\,\mathrm {m^{3}\,kg^{-1}}$ is used for all cases. The time of breakthrough, defined as the time in which the injected solute reaches the right edge of the of the porous domain, is represented in each plot by vertical dotted lines (same color as saturation degrees). The scaling $t^{0.5}$ represents a Fickian regime, respectively, and is shown for visual reference. Time has been non-dimensionalized by the corresponding advective time $t_a$ of each saturation. The continuous lines represent the cases where the adsorption is taking place in the solid grains only, whereas the dashed lines represent when the adsorption is both in the solid grains and at the liquid-gas interface. See Sect. 3.4 for discussion about the impact of adsorption at the liquid-gas interface

Figure 5 shows the evolution in time of the total mass of solute adsorbed in the solid phase over the entire domain $M_\mathrm {s}$  for three different adsorption isotherms, i.e., same coefficient $K_\mathrm {d}$= $1\,\mathrm {m^{3}\,kg^{-1}}$ and three different exponents $\beta =0.2,1.0$, and 2.0, and for all four saturation degrees. A detailed discussion on the impact of the isotherm exponent $\beta$ is presented in Sect. 3.3. For all three analyzed $\beta$-values, an initial increase of $M_\mathrm {s}$ in time is observed for all four $S_\mathrm {w}$ until approximately $t/t_a=1$. The small differences in $M_\mathrm {s}$ obtained at early times for different saturation degrees respond to variations in the surface area available for adsorption due to different spatial distributions of air clusters near the inlet (see Figure S1 of the Supporting Information for details on the phase distributions). Even though at lower $S_\mathrm {w}$ the solute has a much lesser area available to spread (Fig. 3c and f), the mixing area grows similarly in all of them (Fig. 4b). After $t/t_a=1$, the rate of adsorption decreases in all cases, although it remains higher for the saturated case ($\sim t^{0.5}$, and decreasing as $\beta$ increases) than for the unsaturated cases, which contrasts with the opposite trend observed in the temporal growth of the mixing area (Fig. 4b). This behavior can be explained by the larger number of accessible liquid-solid interfaces for $S_{\mathrm {w}}=1.00$ compared to unsaturated cases, and also by the enhanced dilution within the mixing area, as discussed later in this section. Figure 5 also shows the time of solute breakthrough at the right edge of the porous domain, represented by vertical dashed lines for each saturation degree. For all unsaturated cases, $M_\mathrm {s}$ keeps increasing after breakthrough time. This is the strongest for $S_{\mathrm {w}}=0.71$ and can be linked to the larger proportion of stagnation zones in the pore space (Velásquez-Parra et al. 2021), confirming the occurrence of incomplete mixing behind the front, i.e., adsorption keeps occurring specially in the stagnation zones. The final differences in $M_\mathrm {s}$ across saturation degrees observed in the plateaus reached after breakthrough are a consequence of both the mixing dynamics and the difference in surface area available for adsorption to take place.

**Fig. 6:**
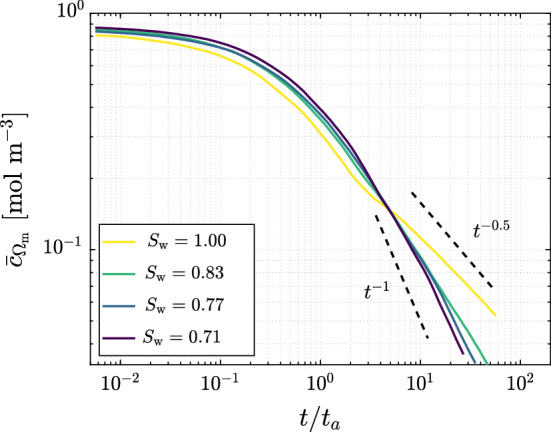
Time evolution of the mean concentration within the mixing volume $\overline{c}_{{\Omega }_\mathrm {m}}$ until solute breakthrough at the right edge of the porous domain for saturation degrees $S_{\mathrm {w}}=1.00,\,0.83,\,0.77$, and 0.71. The dashed lines are shown for reference and represent the scaling of a diffusive system $(t^{-0.5})$ and $(t^{-1})$. Time has been non-dimensionalized by the corresponding advective time $t_a$ of each saturation

To better explain the interplay between mixing and adsorption, we examine the time evolution of the mean concentration within the mixing volume $\overline{c}_{{\Omega }_\mathrm {m}}$, i.e., dilution, for all saturation degrees (Fig. 6). At early times, $\overline{c}_{{\Omega }_\mathrm {m}}$ decreases only slightly and in a very similar way for all four cases, hinting a well mixed system in which fluctuations in the mean concentration are only small (Paster et al. 2015). At approximately $t/t_a=0.4$, $\overline{c}_{{\Omega }_\mathrm {m}}$ starts decreasing regardless of saturation degree, although with slightly different rate in each case. This decrease is explained by the process of dilution, which describes the reduction in the mean solute concentration in the liquid phase as a result of both the mixing process with the resident solution and, to a lesser extent, the adsorption of the solute to the solid phase. At long times, the mean concentration is expected to scale diffusively as $t^{-0.5}$ (Dentz et al. 2011). This behavior is only obtained for $S_\mathrm {w}$ $=1.00$. In contrast, all unsaturated cases dilute faster, showing a scaling that lies somewhere between two regimes, approaching the scaling $\sim t^{-1}$ as $S_\mathrm {w}$ decreases. Although different isotherms contribute differently to the removal of solute mass from the liquid phase (Fig. 5) and therefore to the reduction in the mean concentration $\overline{c}_{{\Omega }_\mathrm {m}}$, for the low environmental concentration considered in this work, the differences in the temporal evolution of dilution between isotherm parameterizations are negligible (and not addressed here for the sake of brevity).

### Impact of the Isotherm Exponent $\beta$ and Coefficient $K_\mathrm {d}$ on Adsorption

Magnitudes for both isotherm parameters were chosen following the batch experiments reported in Botella-Espeso et al. (2021) and Traverso-Soto et al. (2014). They do not report large variations in $\beta$ for the different soil types and adsorbates tested. However, large variations in $K_\mathrm {d}$ do occur. The impact of $\beta$ [0.2-2] (for a single $K_{\mathrm {d}}=1\,\mathrm {m^{3}\,kg^{-1}}$) on the temporal evolution of the absorbed mass $M_\mathrm {s}$ is presented in Fig. 5. An increase in $\beta$ decreases the final value of absorbed mass regardless of saturation degree. This is explained by the functional shape of the isotherm, where for a same $c_{\mathrm {d}}$ a smaller $c_{\mathrm {s}}$ is expected to be adsorbed as $\beta$ increases (Fig. 2). If in addition a more efficient dilution of $c_\mathrm {d}$ occurs, as in unsaturated conditions, the potential for adsorption is further reduced.

**Fig. 7:**
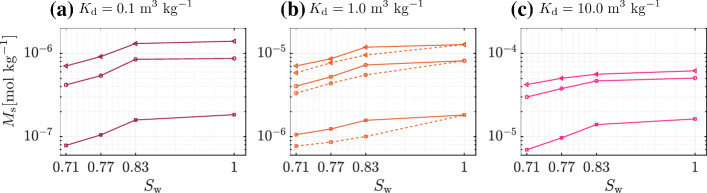
Total mass adsorbed in the solid phase $M_\mathrm {s}$ plotted as a function of the saturation degree $S_\mathrm {w}$ and at same dimensionless time ($t/t_a=38.7$) for **a** $K_{\mathrm {d}}=0.1\,\mathrm {m^{3}\,kg^{-1}}$, **b** $K_{\mathrm {d}}=1\,\mathrm {m^{3}\,kg^{-1}}$, and **c** $K_{\mathrm {d}}=10\,\mathrm {m^{3}\,kg^{-1}}$. In all three subplots, $M_\mathrm {s}$  is shown for three different isotherm exponents: $\beta =2.0$ (squares), 1.0 (circles), and 0.2 (triangles). In panel **b** for $K_{\mathrm {d}}=1\,\mathrm {m^{3}\,kg^{-1}}$ the dashed lines represent the reduction in adsorption to the solid grains when adsorption is occurring at the liquid-gas interfaces along with that in the solid grains

**Fig. 8:**
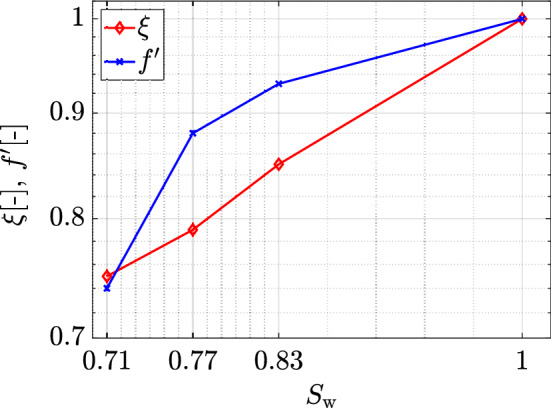
Ratios $\xi$ and $f'$ plotted as a function of saturation. $\xi$ corresponds to the surface area of the liquid-solid interfaces relative to the corresponding value for $S_{\mathrm {w}}=1.00$. $f'$ represents the ratio of the area of preferential paths to total pore space area and is defined as $f'=1-f$, where *f* is ratio of stagnation zones (dead-ends) area to total pore space area. The *y*-axis is plotted in logarithmic scale to allow direct comparison with the trends observed in Figure 7

We perform three different sets of simulations for each pair of $S_\mathrm {w}$ and $\beta$-value. Each set is created by varying $K_\mathrm {d}$ by an order of magnitude at a time, starting from $K_{\mathrm {d}}=0.1\,\mathrm {m^{3}\,kg^{-1}}$ up to $K_{\mathrm {d}}=10\,\mathrm {m^{3}\,kg^{-1}}$ (Fig. 7). All plotted values correspond to the same dimensionless time ($t/t_a=38.7$), chosen shortly before the earliest breakthrough at the right edge of the porous domain, i.e., the one corresponding to $S_\mathrm {w}$ $=0.71$. Overall, results show that the total absorbed mass $M_\mathrm {s}$ increases either with increasing saturation degree $S_{\mathrm {w}}$, with decreasing isotherm exponent $\beta$, or with increasing isotherm coefficient $K_{\mathrm {d}}$. The largest sensitivity in the adsorption response is observed for the latter, as $M_\mathrm {s}$ increases by approximately one order of magnitude as $K_\mathrm {d}$ is increased by a similar factor. This in general occurs for any combination of exponent $\beta$ and $S_\mathrm {w}$, although differences across exponents $\beta$ become less strong for larger values of $K_\mathrm {d}$. Adsorption is highly nonlinear with varying $S_\mathrm {w}$. For all combinations of $K_\mathrm {d}$ and $\beta$, $M_\mathrm {s}$ varies more strongly at the lowest range of saturations, between $S_{\mathrm {w}}=0.71$ and $S_{\mathrm {w}}=0.83$, while the variations tend to flatten out as we reach a fully saturated case. We explain this trend by finding a link with the area in the system available for adsorption. Initially, we compute the ratio $\xi$ of the surface area of the liquid-solid interface at each $S_\mathrm {w}$ to the corresponding area available in the fully saturated case (Fig. 8). The computed values follow a clear linear trend with $S_\mathrm {w}$ for the range of $S_\mathrm {w}$ explored (Culligan et al. 2004), not resembling the behavior shown in Fig. 7. Since the mixing-controlled adsorption is strongly impacted by the heterogeneity of the system, we then resource to an index that accounts for this heterogeneity by describing its effects on the liquid flow velocity field. The index *f* describes the ratio of stagnation zones to the total area of the pore space, thus increasing with decreasing $S_\mathrm {w}$ (Velásquez-Parra et al. 2021). To more explicitly link it with adsorption, which is shown to occur mainly on the preferential paths of high velocity (see Fig. 3), we use $f' = 1-f$ instead, thus showing the ratio of preferential paths to the total pore space area. As observed in Fig. 8, the behavior of $f'$ follows a very similar trend to that shown in Fig. 7 for $M_\mathrm {s}$, where the variation between $S_{\mathrm {w}}=1.00$ and $S_{\mathrm {w}}=0.83$ is very small compared to the change between $S_{\mathrm {w}}=0.83$ and $S_{\mathrm {w}}=0.71$. This helps us conclude on the dominant role of solute spreading on adsorption in unsaturated systems, as it is the main mechanism allowing the solute to reach solid boundaries at time scales where the solute concentration in the liquid phase has not yet been largely reduced by mixing. This contrasts with the lesser adsorption occurring in stagnation zones, as explained in Sect. 3.2 and as shown in Fig. 5, seen in the further small increase of $M_\mathrm {s}$ after breakthrough. We can then establish a direct relationship between geometric characteristics of the liquid flow field, namely the spatial distribution of the liquid flow velocities, and adsorption.

### Impact of adsorption at the liquid-gas interface

Many different kinds of isotherms can be used to model adsorption at liquid-gas interface (Kinniburgh 1986; Borwankar and Wasan 1983). For the sake of brevity, in this work we consider the same adsorption isotherm and kinetics for both the liquid-solid and liquid-gas interfaces. In Fig. 5 we compare the mass adsorbed in the solid grains $M_\mathrm {s}$ for the two cases at the same $K_\mathrm {d}$= $1\,\mathrm {m^{3}\,kg^{-1}}$, first when there is no adsorption at the liquid-gas interface (continuous lines) and second when there is adsorption at the liquid-gas interface (dashed lines). We see a very similar trend for each of the isotherm exponents. The adsorption at the solid grains is larger as $\beta$ reduces. This is consistent with the functional shape of the isotherm. The temporal dynamics are also similar in each case, the key difference being after breakthrough, with a marked reduction in the final total mass adsorbed to the solid phase as seen in the dashed lines. Further in Fig. 7b we compare total mass adsorbed in the solid phase $M_\mathrm {s}$ for $K_\mathrm {d}$= $1\,\mathrm {m^{3}\,kg^{-1}}$. Again, we see the reduction in adsorption in the solid grains when there is adsorption taking place at the liquid-gas interface. Table 1 depicts the length of the liquid-solid interface $\Gamma _{\mathrm {l-s}}$ and liquid-gas interface $\Gamma _{\mathrm {l-g}}$. The ratio $\Gamma _{\mathrm {l-g}}/\Gamma _{\mathrm {l-s}}$ increases as saturation reduces. Therefore, proportionally, less area of solid grains is available for the surfactant to be adsorbed at lower saturation. The gas phase therefore acts as a deterrent to adsorption at the solid phase when saturation is less, although not linearly.

## Conclusions and Outlook

In this study, we aimed to characterize and quantify the impact of fluid-fluid mixing on adsorption at the pore-scale as a function of liquid phase saturation using fluid flow and transport numerical simulations in a 2D porous medium. Using the liquid-phase distribution as an input to the numerical model, we demonstrate the contrasting roles of incomplete mixing and dilution on adsorption to the solid phase. At the same Péclet number, and in addition to a reduction in the solid phase area available for adsorption, a decrease in saturation causes the system to dilute faster, hence reducing faster the solute concentration in the liquid phase. As a consequence, the amount of adsorbed mass is reduced compared to fully saturated conditions. This process is the result of the larger deformation that the solute plume suffers for $S_\mathrm {w}$ $<1.00$, as it is advected by a more heterogeneous velocity field. By increasing the surface in contact with the resident solution, more concentration gradients develop, mainly perpendicular to the main flow direction, thus enhancing mixing. Adding to the effect of dilution, the heterogeneous velocity field also induces incomplete mixing by trapping fractions of solute in stagnation zones, which are diffusion-controlled. Once the solute diffuses its way to the interface with the solid phase inside these regions, a further late increase in the adsorbed mass takes place. The mechanisms described here allow us to explain that the discrepancies commonly found in the adsorption rates between laboratory batch experiments and field scale observations are not only due to a reduction in the surface area for adsorption but also by a dilution driven by the transport processes. They highlight the role that pore-scale processes play on adsorption, commonly neglected on analysis at the Darcy-scale. We also studied the impact of the parameters of the Freundlich isotherm on adsorption. We showed the important role of the coefficient $K_\mathrm {d}$ at controlling the amount of adsorbed mass $M_\mathrm {s}$ in the system, whereas the exponent $\beta$ determines its temporal evolution, especially at late times when the system is highly diluted. We have identified the nonlinear dependency of adsorption on saturation, which seems to be controlled by the heterogeneity of the porous medium and its impact shaping the velocity field in the system. We have also studied the impact of adsorption at the liquid-gas interface on adsorption in the solid grains. A further step in understanding the role that solute mixing and dilution play in these systems could then focus on considering different isotherms and kinetics at the liquid-solid and liquid-gas interfaces. Previous studies have shown that adsorption of surfactants at the liquid-gas interface does not seem to behave linearly as a function of solute concentration (Yan et al. 2020), adding further complexity to this research question. The effect of small Damköhler numbers (adsorption kinetics are faster than mixing) on adsorption should also be further studied, since they might enhance the occurrence of incomplete mixing across the system. We believe several of our observations to hold for 3D systems as well. Similar air cluster size distributions (Iglauer et al. 2010, 2012; Scheffer et al. 2021) and a similar flow structure, displaying a clear separation of liquid flow in stagnation regions and preferential paths (Holzner et al. 2015), have also been described in three-dimensional conditions. It has also been shown previously that the mixing dynamics in 3D unsaturated porous media follow the same scaling laws as in 2D unsaturated flows (Markale et al. 2021). Therefore, we expect the adsorption mechanisms we describe for these 2D flow structures to remain valid for 3D conditions. The existence of a third degree of freedom for liquid flow to happen might also lead to twisted streamlines (Ye et al. 2015) and favor chaotic advection (Lester et al. 2013), conditions under which mixing is enhanced. This might further enhance dilution in the liquid phase, as some studies have suggested (Heyman et al. 2021). We believe that our results are a significant step in understanding the mechanisms controlling adsorption in unsaturated systems, and represent an important contribution for assessing the fate of contaminants traveling in such conditions.

## Supplementary Information

Below is the link to the electronic supplementary material.

Supplementary file 1 (mp4 6821 KB)

Supplementary file 2 (mp4 7995 KB)

Supplementary file 3 (mp4 7968 KB)

Supplementary file 4 (mp4 7303 KB)

Supplementary file 5 (mp4 2369 KB)

Supplementary file 6 (mp4 2983 KB)

Supplementary file 7 (mp4 3010 KB)

Supplementary file 8 (mp4 2698 KB)

Supplementary file 9 (pdf 5528 KB)

## Data Availability

The data that support the findings of this study are available from the corresponding author upon reasonable request.

## Code Availability

Numerical flow and reactive transport simulations were performed using the software COMSOL Multiphysics. The code used to post-process the numerical results and to produce the figures is a tailored MATLAB script.
