# Supplementary material for: Mixing Controlled Adsorption at the Liquid-Solid Interfaces in Unsaturated Porous Media
Source: Transp Porous Media. 2022 Feb 5;146(1-2):159–75. doi: 10.1007/s11242-022-01747-x (PMC9849304; doi:10.1007/s11242-022-01747-x)
Supplement: Supplementary file 9 — (pdf 5528 KB) [file 11242_2022_1747_MOESM9_ESM.pdf]

Transport in Porous Media

Supporting Information:

Mixing Controlled Adsorption at the Liquid-Solid Interfaces in Unsaturated Porous Media

Ishaan Markale[1,2] · **Andrés Velásquez-Parra**[1,2] ·
Andrés Alcolea[3] · **Joaquín Jiménez-Martínez**[1,2]

Contents of this file

- Velocity field for the different saturation degree analyzed.
- Caption for movies M1-M4: Temporal evolution of concentration in the liquid phase and of adsorption in the solid phase.
- Caption for movies M5-M8: Temporal evolution of the mixing area A_{m}.

Additional supporting information - files uploaded separately

- Movies M1-M4
- Movies M5-M8

1 Velocity field as function of saturation degree

Figure S1 shows the distribution of phases and the velocity field in the liquid phase obtained from Stokes flow numerical simulations for the four different saturation degrees analyzed in our study. The logarithm of velocities is plotted. The flow occurs from left to right. The color scale is common to all panels, with red color indicating higher velocities and blue color showing the lower end of velocities. The flow field in the saturated case ($S_{\mathrm{w}} = 1.00$) shows limited variability (Fig. S1a). This increases largely at lower S_{w} and is depicted in the strong enhancement of high velocity regions (referred to as preferential paths) and low

J. Jiménez-Martínez

E-mail: joaquin.jimenez@eawag.ch / jjimenez@ethz.ch

[1] Department of Water Resources and Drinking Water, Eawag, 8600 Dübendorf, Switzerland

[2] Department of Civil, Environmental and Geomatic Engineering, ETH Zurich, 8093 Zürich, Switzerland

[3] HydroGeoModels AG, Tösstalstrasse 23, 8400 Winterthur, Switzerland

velocity regions (referred to as stagnation zones). In the regions of high velocity magnitudes, we observe a higher level of adsorption, as also shown in the Movies M1-M4.

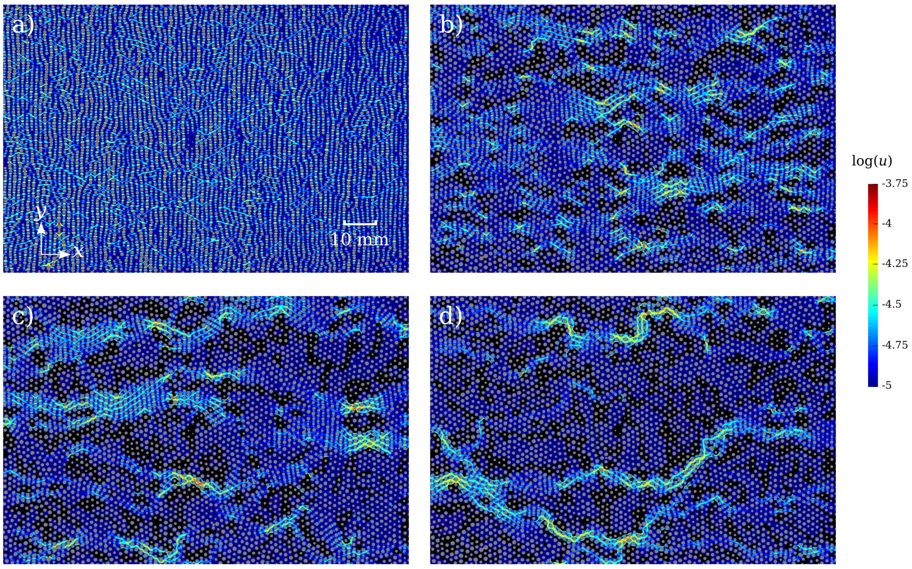

Fig. S 1. Spatial distribution of steady-state velocities in the liquid phase for liquid phase saturation a) S_w=1.00, b) S_w=0.83, c) S_w=0.77, and d) S_w=0.71. The black color represents air clusters, while solid obstacles are shown in gray. The rainbow color bar on the right is common for all subfigures, depicting high velocities in red color and low velocities in blue tones.

2 Caption movies M1-M4: Temporal evolution of concentration in the liquid and adsorption in the solids

Spatio-temporal evolution of both concentration in the liquid phase and adsorption in the solid phase, for all four analyzed saturation degrees M1: $S_w = 1.00$, M2: $S_w = 0.83$, M3: $S_w = 0.77$, and M4: $S_w = 0.71$. Note that the time evolution is shown in logarithmic scale, i.e., the time lapse between two consecutive frames at early times is smaller compared to later times. Red tones indicate higher concentrations, while blue tones depict lower concentrations.

3 Caption movies M5-M8: Temporal evolution of the mixing area A_m

Spatio-temporal evolution of the mixing area for all saturation degrees M5: $S_w = 1.00$, M6: $S_w = 0.83$, M7: $S_w = 0.77$, and M8: $S_w = 0.71$. The mixing area A_m is shown in yellow color, while the rest of the pore space is shown in blue color. Note that the time evolution is shown in logarithmic scale, i.e., the time lapse between two consecutive frames at early times is smaller compared to later times.
